# Novel oral anticoagulants in primary care in patients with atrial fibrillation: a cross-sectional comparison before and after their introduction

**DOI:** 10.1186/s12875-018-0796-4

**Published:** 2018-07-18

**Authors:** Simon Schwill, Katja Krug, Frank Peters-Klimm, Jan van Lieshout, Gunter Laux, Joachim Szecsenyi, Michel Wensing

**Affiliations:** 1 Department of General Practice and Health Services Research, University Hospital Heidelberg, Heidelberg, Germany; 2 Radboud Institute of Health Sciences, Radboud University Medical Centre, Nijmegen, Netherlands

**Keywords:** Atrial fibrillation, Stroke prevention, Oral anticoagulation, Vitamin K antagonists, Novel oral anticoagulants

## Abstract

**Background:**

Novel oral anticoagulation (NOAC) has been introduced in recent years, but data on use in atrial fibrillation (AF) in primary care setting is scarce. In Germany, General Practitioners are free to choose type of oral anticoagulation (OAC) in AF. Our aim was to explore changes in prescription-rates of OAC in German primary care before and after introduction of NOAC on the market.

**Methods:**

Data of a representative morbidity registration project in primary care in Germany (CONTENT) were analysed. Patients with AF in 2011 or 2014 were included (before and after broad market authorization of NOAC, respectively). We defined three independent groups: patients from 2011 without follow-up (group A), patients from 2014 but without previous record in 2011 (group B) and patients with AF and records in 2011 and 2014 (group C).

**Results:**

2642 patients were included. Group A (*n* = 804) and B (*n* = 755) were comparable regarding patient characteristics. 87.3% of group A and 84.8% of group B had CHA_2_DS_2_-VASc-Score ≥ 2, indicating a need for oral anticoagulation (OAC). Prescription of OAC increased from 23.1% (*n* = 186) to 42.8% (*n* = 323, *p* < .01) with stable use of vitamin-k-antagonist (22.6–24.9%). NOAC increased from 0.6 to 19.2% (p < .01). Monotherapy with Acetylsalicylic acid (ASA) decreased from 15.3% (*n* = 123) to 8.2% (*n* = 62, p < .01). In group C (*n* = 1083), OAC increased from 35.3 to 55.4% (p < .01), with stable prescription rate of vitamin-k-antagonist (34.4–35.7%). NOAC increased from 0.9 to 21.5% (p < .01).

**Conclusions:**

In summary, our study showed a significant increase of OAC over time, which is fostered by the use of NOAC but with a stable rate of VKA and a sharp decrease of ASA. Patients on VKA are rarely switched to NOAC, but new patients with AF are more likely to receive NOAC.

## Background

Atrial fibrillation (AF) is the most common cardiac arrhythmia, with an estimated prevalence of 1.5 to 2% of the general population, which is increasing worldwide, driven by an ageing population [1–3]. Nearly 10% of people above 75 years suffer from AF [4]. Patients have an almost fivefold higher risk of stroke [5] and mortality is up to 2.5 times higher compared with age-matched individuals [2].

Regardless of causal treatment, prophylaxis of stroke by use of oral anticoagulation (OAC) was shown to reduce risk of stroke and therefore is recommend in patients with AF by international guidelines [6, 7]. The CHA_2_DS_2_-VASc-Score has been recommended for risk stratification [6–8]. For decades vitamin-K antagonists (VKA) had been the only OAC in AF, accompanied by prescription of acetylsalicylic acid (ASA), which is no longer recommended [6, 7]. In recent years novel-oral-anticoagulants (NOAC) such as Apixaban, Dabigatran, Edoxaban and Rivaroxaban offered an alternative OAC to prevent stroke in non-valvular AF. Use of NOAC is increasing and recently updated guidelines on AF by the European Society of Cardiology advocate use of NOAC over VKA [6]. Clinical guidelines on AF for primary care physicians present NOAC as a valid option, but do not necessarily recommend changing patients from VKA to NOAC [9].Data on use of (N)OAC in AF in routine primary care setting is scarce [10–14]. In Germany, NOAC were licensed for the indication of oral anticoagulation for the prevention of stroke in non-valvular atrial fibrillation between November 2011 and June 2015: Dabigatran in 11 /2011, Rivaroxaban in 12/2011, Apixaban in 12/2012 and Edoxaban in 06/2015 [15–18]. The aims of this study were to document changes in prescription-rates of OAC (VKA and NOAC) in primary care patients with AF in Germany before and after introduction of NOAC on the market, and to explore whether known patients with AF were switched onto NOAC.

## Methods

### Study design

We performed a retrospective epidemiological study in primary care practices including a cross-sectional comparison of two independent samples and a cross-sectional comparison of same patients at two points of time.

### Setting

Data were derived from the CONTinuous-morbidity-registration-Epidemiologic-NeTwork (CONTENT) in South-Germany, which started in 2005 [19]. A total of 43 general practices were involved in this study. CONTENT provides representative data in terms of social, ethnic and economic backgrounds as well as urban, suburban and rural areas. The quality of data is high due to constant education of participating teams as well as continuous feedback on recording quality to participating physicians.

### Sample

In 2016, data of CONTENT starting in 2005 up to 31.12.2014 was included in the study and analysed retrospectively. We performed a before and after comparison on the prescription of OAC, which compared data from 2011, when there was little experience with NOAC and data from 2014, when Apixaban, Dabigatran and Rivaroxaban were broadly available. Edoxaban was not licensed in Germany in 2014. Out of all patients with diagnosed AF (ICD I48.0) we included patients with AF from 01.01.2011–31.12.2011 or 01.01.2014–31.12.2014. Afterwards, we, defined three independent groups: patients with AF in 2011 but without follow-up in 2014(group A), patients with AF in 2014 but without previous record in 2011 (group B) and patients with AF and records in 2011 and 2014 (group C). Difference between groups A and B was related to newly diagnosed AF, mortality and patient change of practice. Patients of group C were not included in other groups neither A nor B.

### Measures

Relevant data were extracted from the CONTENT database. For each patient age, gender as well as prescription of medication, diagnoses and specialist referrals were determined. Age was calculated from the year of birth. Gender and age were used for measurement of CHA_2_DS_2_-VASc-Score. Diagnoses were given as ICD-10 codes. Prescriptions were given as ATC-codes (Anatomical Therapeutical Classification for medication, World Health Organization). Secondly, prescriptions were analysed for VKA [Phenprocoumon = ATC-B01AA04] and NOAC [Apixaban = ATC-B01AX08, Dabigatran = ATC-B01AE07, Rivaroxaban = ATC-B01AX06] and ASA [acetylsalicylic-acid = ATC-B01AC06]. We extracted diagnoses needed for the CHA_2_DS_2_-VASc-Score: Congestive heart failure (I50.*), Hypertension (I10.*) Diabetes (E10.-E11.*, E14.*), Stroke (I63.*) and Vascular disease (I70.*). At time of study CHA_2_DS_2_-VASc-Score ≥ 2 indicated OAC in patients with AF^7^. If data of hypertension or diabetes were not encoded, prescription of an antidiabetic [ATC-A10] or antihypertensive [angiotensin-converting-enzyme-inhibitor = ATC-C09AA, Angiotensin-II-receptor-antagonists = ATC-C09CA, Nifedipine = ATC-C08CA05, Hydrochlorothiazide = ATC-C03AA03] medication was accepted instead.

To address potential contraindications we extracted diagnoses renal insufficiency (N17.*-N19.*), coagulopathy (D68.-D69.*), intracranial bleeding (I60.-I62.*, S06.4-S06.6.*, S06.9), epistaxis (R04.0-R04.2), gastrointestinal bleeding (K92.0-K92.2, K62.5) and stroke (I63.-I64.*). We recorded specialist referrals only to cardiologists.

### Data analysis

Patient-characteristics were summarized in terms of frequencies of categories, means with standard deviation and medians with interquartile range for continuous variables. Differences between independent groups A and B were tested in Chi-squared-tests for frequencies, Fishers exact test, t-tests for independent samples or MannWhitney-U-tests for continuous data, dependent on data distribution. Changes over time (group C) were analysed with McNemar’s-tests for frequencies and t-tests for dependent samples or Wilcoxon-signed-rank-tests for continuous data, dependent on data distribution. *P*-values < 0.05 were considered to indicate statistical significance. Due to the exploratory character of the analysis, there was no correction for multiple testing. All analyses were performed with IBM SPSS version 21.0.

## Results

### Patient characteristics

Total number of patient in CONTENT in 2011 was *n* = 145,461 (point-prevalence of AF on 31.12.2011: 1.51%), in 2014 *n* = 207,253 (point-prevalence of AF on 31.12.2014: 1.85%).In total, data of *n* = 2642 patients from 43 general practices was obtained in this study. We included *n* = 804 in Group A, *n* = 755 in Group B and *n* = 1083 in Group C. In two-sample comparison, presented in Table 1, groups A and B did not differ regarding sex or comorbidities, but group B was younger than group A (*p* < 0.01). Number of patients > 75 years, contributing to CHA_2_DS_2_-VASc-Score twice, was 66.3% in group A (*n* = 533) which was comparable to 61.9% in group B (*n* = 467). 87.3% of patients in group A (*n* = 702) and 84.8% of patients in group B (*n* = 640) presented with CHA_2_DS_2_-VASc-Score ≥ 2, which indicated OAC at time of the study (p = n.s.).

**Table 1:** Patient characteristics comparing 2011 and 2014 independently

			GROUP A (2011)	GROUP B (2014)	*p*-value
patients (n)	total		804	755	
	gender (%)	male	374 (46.5%)	388 (51.4%)	.06^a^
		female	429 (53.4%)	367 (48.6%)	
		undetermined	1 (0.1%)	0	
	age (years)	Median (IQR)	79 (71–86)	77 (70–83)	<.01^b^
		Min – Max	19–99	18–103	
		< 65	92 (11.4%)	118 (15.6%)	
		65–74	179 (22.3%)	170 (22.5%)	
		≥75	533 (66.3%)	467 (61.9%)	
	additional diagnosis	at least 1 additional	750 (93.3%)	724 (95.9%)	.02^a^
		renal insufficiency	54 (6.7%)	55 (7.3%)	.66^a^
		coagulopathy	13 (1.6%)	17 (2.3%)	.36^a^
		intracranial bleeding	8 (1.0%)	6 (0.8%)	.68^a^
		epistaxis	7 (0.9%)	10 (1.3%)	.39^a^
		gastrointestinal bleeding	15 (1.9%)	12 (1.6%)	.68^a^
		stroke	43 (5.3%)	34 (4.5%)	.44^a^
	CHA_2_DS_2_-VASC:	0	48 (6.0%)	56 (7.4%)	.34^a^
		1	54 (6.7%)	59 (7.8%)	
		≥2	702 (87.3%)	640 (84.8%)	
	prescriptions (per year)	patients with at least 1 prescription	684 (85.1%)	677 (89.7%)	<.01^a^
		Md (IQR)	12 (5–25)	15 (6–24)	.31^b^
		Min - Max	1–123	1–123	
		all patients			.02^b^
		Md (IQR)	10 (2–22.8)	13 (3–23)	
		Min - Max	0–123	0–123	
	OAC	VKA or NOAC	186 (23.1%)	323 (42.8%)	<.01^a^
		VKA	182 (22.6%)	188 (24.9%)	.29^a^
		Rivaroxaban	0 (0.0%)	121 (16.0%)	<.01^a^
		Dabigatran	5 (0.6%)	14 (1.9%)	.03^a^
		Apixaban	0 (0.0%)	10 (1.3%)	<.01^c^
	ASA (without additional OAC)^d^		123 (15.3%)	62 (8.2%)	<.01^a^
	consultation of cardiologist		125 (15.5%)	165 (21.9%)	<.01^a^

CONTENT-Patients with diagnosis of atrial fibrillation, which were registered only once, either in 2011 (group A) or in 2014 (group B). *OAC* oral anticoagulation, *VKA* vitamin-k antagonists, *NOAC* novel oral anticoagulation, *ASA* Acetylsalicylic acid

^a^Chi^2^-test, ^b^Mann-Whitney-U-test, ^c^Fisher’s exact test

^d^Indication for ASA (100 mg), such as peripheral vascular disease or post-stroke or post-myocardial infarction or else and either as primary or secondary prophylaxis, could not clearly be stated

Group C included n = 1083 patients (Table 2), of whom 51.9% were above 75 years in 2011 (*n* = 562) and 64.4% in 2014 (*n* = 697). Percentage of patients with CHA_2_DS_2_-VASc-Score ≥ 2 increased from 81.6% (*n* = 884) to 83.8% (*n* = 908, *p* < .01). 91% of patients with diagnosed AF presented with at least one additional diagnosis, such as renal insufficiency (*n* = 1003).

**Table 2:** Patient characteristics over time

			GROUP C (2011)	GROUP C (2014)	*p*-value
patients (n)	total		1083		n/a
	gender (%)	male	584 (53.9%)		n/a
		female	470 (43.4%)		
		undetermined	29 (2.7%)		
	age (years)	Median (IQR)	75 (68–81)	78 (71–84)	n/a
		Min – Max	24–93	27–96	
		< 65	200 (18.5%)	145 (13.4%)	
		65–74	321 (29.6%)	241 (22.3%)	
		≥75	562 (51.9%)	697 (64.4%)	
	additional diagnosis	patients With at least 1	1003 (92.6%)	986 (91.0%)	.13^a^
		renal insufficiency	61 (5.6%)	65 (6.0%)	.77^a^
		coagulopathy	17 (1.6%)	17 (1.6%)	1.0^a^
		intracranial bleeding	4 (0.4%)	5 (0.5%)	1.0^a^
		epistaxis	21 (1.9%)	14 (1.3%)	.30^a^
		gastrointestinal bleeding	13 (1.2%)	17 (1.6%)	.59^a^
		stroke	33 (3.0%)	28 (2.6%)	.58^a^
	CHA_2_DS_2_-VASC:	0	92 (8.5%)	59 (5.4%)	<.01^a^
		1	100 (9.2%)	87 (8.0%)	
		≥2	884 (81.6)	908 (83.8%)	
		not computable	7 (0.6%)	29 (2.7%)	n/a
	prescriptions (per year)	total	1056 (97.5%)	1064 (98.2%)	n/a
		M (SD)	19.4 (14.6)	22.6 (16.5)	<.01^b^
		Min - Max	1–118	1–115	
		all patients including those without prescription			<.01^b^
		M (SD)	18.8 (14.8)	22.0 (16.7)	
		Min - Max	0–118	0–115	
	OAC^1^	VKA or NOAC	382 (35.3%)	600 (55.4%)	<.01^a^
		VKA	373 (34.4%)	387 (35.7%)	.41^a^
		Rivaroxaban	3 (0.3%)	181 (16.7%)	<.01^a^
		Dabigatran	7 (0.6%)	23 (2.1%)	<.01^a^
		Apixaban	none	28 (2.6%)	n/c
	ASA (without additional OAC)^2^		136 (12.6%)	97 (9.0%)	<.01^a^
	consultation of cardiologist		245 (22.6%)	262 (24.2%)	.32^a^

CONTENT-Patients with diagnosis of atrial fibrillation, which were observed over time including data from 2011 and follow-up in 2014 (group C). OAC = oral anticoagulation, VKA = vitamin-k antagonists, NOAC = novel oral anticoagulation, ASA = Acetylsalicylic acid

^1^Different prescriptions per year such as VKA and NOAC or NOAC and NOAC was possible (*n* = 29)

^2^Indication for ASA (100 mg), such as peripheral vascular disease or post-stroke or post-myocardial infarction or else and either as primary or secondary prophylaxis, could not clearly be stated

^a^McNemar test, ^b^ t-test for dependent samples

### Prescription of oral-anticoagulation

Two-sample comparison revealed an increase of prescription of OAC from 23.1% in 2011 (*n* = 186) to 42.8% in 2014 (*n* = 323, *p* < .01). Prescription-rate of VKA remained stable (22.6%, *n* = 182 and 24.9%, *n* = 188). Prescription of NOAC increased from 0.6% (n = 5) to 19.2% (*n* = 145, *p* < .01), whereas monotherapy with ASA decreased from 15.3% (*n* = 123) to 8.2% (*n* = 62, *p* < .01). The majority of patients with NOAC in 2014 received Rivaroxaban (84.8%, *n* = 121) compared to Dabigatran (9.6%, n = 14) and Apixaban (6.9%, n = 10). In 2014, cardiologists were involved more often compared with 2011 (15.5%, *n* = 125versus 21.9%, *n* = 165, *p* < .01).

In group C (involving longitudinal comparison, *n* = 1083), prescription of OAC increased from 35.3% (*n* = 382) to 55.4% (*n* = 600, p < .01) with similar prescription rate of VKA (34.4%, *n* = 373 and 35.7%, *n* = 387). Mean annual prescriptions per patient increased from 19.3 to 22.4 (p < .01). Prescription of NOAC in group C increased from 0.9 to 21.5% (p < .01). Prescription of ASA decreased over time (2011:12.6%, *n* = 136, 2014:9.0%, *n* = 97, p < .01). 11.9% (*n* = 44) of patients with VKA were switched to NOAC and in 19.9% OAC (*n* = 74) was discontinued completely. In 2011, 9 patients received a NOAC, of which 4 were stopped until 2014 and one patient was switched to VKA. 11.9% (n = 44) of patients with VKA were switched to NOAC. In 2014, majority of patients with AF and OAC received VKA: Group B: 56% *n* = 188/333, Group C: 62.5% *n* = 387/619.

In group C, 22.6–24.2% of patients attended a cardiologist but involvement of cardiologists did not significantly change over time (2011: 22.6%, 2014: 24.2%, *p* < .32). If cardiologists were involved, prescription of OAC increased over time from 51.6% in 2011 to 67.2% of patients in 2014 (*p* < .01).

## Discussion

In patients with recorded AF in German primary care there was in increase in prescription-rate for OAC between 2011 and 2014: A cross-sectional comparison revealed that in 2014 42.8% (*n* = 323) of patients with AF received OAC compared to 23.1% (*n* = 186) in 2011. A longitudinal comparison (group C) showed an increase of OAC in AF from 35.3% (*n* = 383) to 55.4% (*n* = 600). This increase in prescription-rate was mainly due to new prescription of NOAC, while prescription-rate of VKA remained stable. In longitudinal comparison (group C) use of NOAC increased to 21.4% (*n* = 232) of patients with AF in 2014, with Rivaroxaban as most frequently prescribed. In 2014, cardiologists were involved in about 25% of patients (Group B: 21.9% (*n* = 165) and Group C: 24.2% (*n* = 262) and their involvement increased the likelihood of OAC prescribing. However, analysis showed no difference in cardiologists preference between VKA and NOAC and in 2014 majority of patients with OAC in AF still received VKA (Group B: 56% *n* = 188/333, Group C: 62.5% *n* = 387/619). Our analysis showed that patients were rarely switched from VKA to NOAC but were more likely to receive NOAC if diagnosed newly.

Interestingly, over 80% of patients with AF (group A 87.3%, group B 84.8%, group C 81.6%/83.8%) presented with CHADS-VasC Score ≥ 2 indicating OAC (at time of study). High percentage might be explained by the age, as in our study about two third of patients were 75 years of age or higher, scoring 2 within the CHA_2_DS_2_-VASc-Score, and thereby had a formal indication for OAC at time of the study. However, this represents the population in primary care.Recently updated guidelines on AF postulate evidence that OAC can prevent majority of ischemic strokes in AF and prolong life [6]. Although our findings suggest suboptimal prescribing-rates of OAC, they should be interpreted carefully. Patient populations in clinical trials of OAC differ regarding age, sex and co-morbidities from those treated in primary care [20, 21].

The benefit-risk-ratio of OAC and NOAC more particularly, may be less positive in primary care patients compared to patients in cardiology trials. In routine data, we miss documentation of reasons for not-prescribing OAC such as increased bleeding risk, alcohol-abuse, liver-failure or uncontrolled hypertension (which could not be accessed). Qualitative studies on prescribing OAC in primary care are rare, but it can be assumed that low life expectancy, high risk of falling and bleeding, as well as frailty are reasons to refrain from prescribing OAC in AF. This contrasts with views of experts, such as the European-Primary-Care-Cardiovascular-Society (EPCCS), who claim that a high bleeding-risk-score (such as HAS-BLED, ORBIT or ABC) should generally not result in withholding OAC [6, 22].

Our data showed a significant increase in the prescription-rate of OAC in AF before and after market-share of NOAC. The design of our study does not allow causal attribution so it is not known if this increase might be caused by an alternative medication to VKA general practitioners had awaited, by an update and implementation of guidelines on non-valvular AF, by a generous promotion of NOAC or a mix of it all. We showed majority of patients with AF and OAC in 2014 received VKA. Until today, whether NOAC or VKA are safer and/or more effective in prophylaxis of stroke in non-valvular AF remains a key-point of experts-discussions as well as on-going trials [23]. In summary, there is a need for further research into the determinants of prescribing OAC in patients with AF in primary care.

### Strengths and limitations

Strengths of this study are large number of primary care practices and reasonably high quality of patient records. Participating practices had university affiliation, which is associated with higher involvement in continuous medical education. It may be noted that in the German health care system documentation of AF (ICD48.*) must be confirmed by Electrocardiogram (ECG), as recommend in updated guidelines on AF [6]. All participating practices were well experienced in daily use of ECG, but we could not independently verify the recorded diagnosis of AF. Secondly, due to routine data out of patient charts underreporting of AF (ICD48.*) as well as comorbidities and follow-up might be possible [24]. However, we focussed on the prescriptions of OAC for patients with AF and those with undocumented AF cannot be considered for treatment. Finally, we did not know which patients did not receive anticoagulants due to (relative or absolute) contraindications such as liver dysfunction.

## Conclusions

We aimed to document prescription-rates of OAC (VKA and NOAC) in primary care patients with AF in Germany before and after broad market-share of NOAC and to explore whether known patients with AF were switched onto NOAC. In summary, our study showed a significant increase of OAC over time, which is fosterer by the use of NOAC but with a stable rate of VKA and a sharp decrease of ASA. Patients on VKA are rarely switched to NOAC, but new patients with AF are more likely to receive NOAC. In 2014, 45% of patients with CHA2DS2-VASc-Score ≥ 2 were not prescribed OAC, suggesting room for improvement. Further research is needed to identify factors contributing to this.

## Abbreviations

AF: Atrial fibrillation
ASA: Acetylsalicylic acid
CHA2DS2-VASc: Congestive heart failure, Hypertension, Age, Diabetes, Stroke, Vascular Disease, Age, Sex category
CONTENT: CONTinuous-morbidity-registration-Epidemiologic-NeTwork
ECG: Electrocardiogram
ICD: International Statistical Classification of Diseases and Related Health Problems
NOAC: Novel oral anticoagulation
OAC: Oral anticoagulation
VKA: Vitamin-K antagonists
